# Determinants of Judgments of Explanatory Power: Credibility, Generality, and Statistical Relevance

**DOI:** 10.3389/fpsyg.2017.01430

**Published:** 2017-09-04

**Authors:** Matteo Colombo, Leandra Bucher, Jan Sprenger

**Affiliations:** ^1^Tilburg Center for Logic, Ethics and Philosophy of Science, Tilburg University Tilburg, Netherlands; ^2^General and Biological Psychology, University of Wuppertal Wuppertal, Germany

**Keywords:** explanation, prior credibility, causal framing, generality, statistical relevance

## Abstract

Explanation is a central concept in human psychology. Drawing upon philosophical theories of explanation, psychologists have recently begun to examine the relationship between explanation, probability and causality. Our study advances this growing literature at the intersection of psychology and philosophy of science by systematically investigating how judgments of explanatory power are affected by (i) the prior credibility of an explanatory hypothesis, (ii) the causal framing of the hypothesis, (iii) the perceived generalizability of the explanation, and (iv) the relation of statistical relevance between hypothesis and evidence. Collectively, the results of our five experiments support the hypothesis that the prior credibility of a causal explanation plays a central role in explanatory reasoning: first, because of the presence of strong main effects on judgments of explanatory power, and second, because of the gate-keeping role it has for other factors. Highly credible explanations are not susceptible to causal framing effects, but they are sensitive to the effects of normatively relevant factors: the generalizability of an explanation, and its statistical relevance for the evidence. These results advance current literature in the philosophy and psychology of explanation in three ways. First, they yield a more nuanced understanding of the determinants of judgments of explanatory power, and the interaction between these factors. Second, they show the close relationship between prior beliefs and explanatory power. Third, they elucidate the nature of abductive reasoning.

## Introduction

Explanation is a central concept in human psychology. It supports a wide array of cognitive functions, including reasoning, categorization, learning, inference, and decision-making (Brem and Rips, 2000; Keil and Wilson, 2000; Keil, 2006; Lombrozo, 2006). When presented with an explanation of why a certain event occurred, of how a certain mechanism works, or of why people behave the way they do, both scientists and laypeople have strong intuitions about what counts as a good explanation. Yet, more than 60 years after philosophers of science began to elucidate the nature of explanation (Craik, 1943; Hempel and Oppenheim, 1948; Hempel, 1965; Carnap, 1966; Salmon, 1970), the determinants of judgments of explanatory power remain unclear.

In this paper, we present five experiments on factors that may affect judgments of explanatory power. Motivated by a large body of theoretical results in epistemology and philosophy of science, as well as by a growing amount of empirical work in cognitive psychology (for respective surveys, see Lombrozo, 2012; Woodward, 2014), we examine how judgments of explanatory power are affected by (i) the prior credibility of an explanatory hypothesis, (ii) the causal framing of the hypothesis, (iii) the perceived generalizability of the explanation, and (iv) the statistical relevance between hypothesis and evidence.

Specifically, we set out to test four hypotheses. First, we hypothesized that the prior credibility of a causal explanation predicts judgments of explanatory power. Throughout all five experiments, we manipulated the prior credibility of different explanations, and examined the effects of this manipulation on explanatory judgments. We also wanted to understand how low and high prior credibility interacted with other possible psychological determinants of explanatory power.

Our focus on the prior credibility of causal explanation was motivated by the fact that most philosophical and psychological analyses of explanatory power agree that powerful explanations provide information about credible causal relationships (Salmon, 1984; Lewis, 1986; Dowe, 2000). Credible causal information facilitates the manipulation and control of natural phenomena (Pearl, 2000; Woodward, 2003; Strevens, 2008) and plays distinctive roles in human psychology (Lombrozo, 2011; Sloman and Lagnado, 2015). For example, credible causal information guides categorization (Carey, 1985; Murphy and Medin, 1985; Lombrozo, 2009), supports inductive inference and learning (Holyoak and Cheng, 2011; Legare and Lombrozo, 2014; Walker et al., 2014), and calibrates metacognitive strategies involved in problem-solving (Chi et al., 1994; Aleven and Koedinger, 2002).

While the prior credibility of an explanation may be an important determinant of explanatory power, in previous research we found that prior probabilities of candidate explanatory hypotheses had no impact on explanatory judgment when they were presented as objective, numerical base rates (Colombo et al., 2016a), which was consistent with the well-documented phenomenon of base rate neglect (Tversky and Kahneman, 1982). Thus, we decided to focus on the *subjective* prior credibility of an explanation in the present study, in order to better evaluate the effects of prior credibility on explanation.

Our second, related hypothesis was that presenting an explanatory hypothesis in causal terms predicts judgments of its explanatory power. Thus, we wanted to find out whether people's explanatory judgments are sensitive to causal framing effects.

The importance of this issue should be clear in the light of the fact that magazines and newspapers very often, even when it's not warranted, describe scientific explanations in terms of causal language (e.g., “Processed meat causes cancer” or “Economic recession leads to xenophobic violence”) with the aim of capturing readers' attention and boosting their sense of understanding (Entman, 1993; Scheufele and Scheufele, 2010). Thus, Experiments 1 and 2 examined the impact and interaction of prior credibility and causal framing on judgments of explanatory power.

With Experiment 3, we tested the hypothesis that the perceived generalizability of an explanation influences explanatory power. Specifically, in our experiments, we operationalized “generalizability” in terms of the *size of a sample* involved in a study, since the sample size is an obvious, crucial feature of any study in which the aim is to make inferences about a population from a sample. Thus, in our experiments, we aimed to isolate the effects of the perceived generalizability of an explanation, operationalized in terms of sample size, on judgments of explanatory power and its interaction with the prior credibility of an explanation, while controlling for causal framing and statistical relevance.

While the generalizability of scientific results is an obvious epistemic virtue that figures in the evidential assessments made by scientists, it is less clear how lay people understand and use this notion in making explanatory judgments. Previous psychological findings about the role of generalizability in explanatory reasoning are mixed and rely on different operationalizations of generalizability. Read and Marcus-Newhall (1993) operationalized generality in terms of the number of facts that an explanation can account for. For example, given the facts that Silvia has an upset stomach and that Silvia has been gaining weight lately, the explanation that Silvia is pregnant is more general than the explanation that Silvia has stopped exercising. With this operationalization in place, Read and Marcus-Newhall (1993) found that generalizability predicted explanatory judgments. Preston and Epley (2005) understood generalizability in terms of the number of implications or observations that a research finding would explain. They showed that hypotheses that would explain a wide range of observations were judged as more valuable. However, these studies involved no uncertainty about whether or not a causal effect was actually observed (cf., Khemlani et al., 2011), and they did not examine different ways in which people might understand when a hypothesis is generalizable.

With Experiments 4 and 5, we tested our fourth and final hypothesis: that the statistical relevance of a hypothesis for a body of observed evidence is another key determinant of judgments of explanatory power.

According to several philosophers, the power of an explanation is manifest in the amount of statistical information that an *explanans* H provides about an *explanandum* E, given some class or population S. In particular, it has to be the case that Prob (E|H&S) > Prob (E|S) (Jeffrey, 1969; Greeno, 1970; Salmon, 1970). Suppose, for example, that Jones has strep infection, and his doctor gives him penicillin. After Jones has taken penicillin, he recovers within 1 week. When we explain why Jones recovered, we usually cite statistically relevant facts, such as the different recovery rates among treated and untreated patients.

Developing this idea, several research groups have put forward probabilistic measures of explanatory power (McGrew, 2003; Schupbach and Sprenger's, 2011; Crupi and Tentori, 2012). Their approach is that a hypothesis is the more explanatorily powerful the less surprising it makes the observed evidence. Results from experimental psychology confirm this insight. Schupbach (2011) provided evidence that Schupbach and Sprenger's (2011) probabilistic measure is an accurate predictor of people's explanatory judgments in abstract reasoning problems (though see Glymour, 2015). Colombo et al. (2016a) found that explanatory judgments about everyday situations are strongly affected by changes in statistical relevance. Despite these results, it remains unclear how statistical relevance interacts with other factors to determine explanatory power, in particular the prior credibility of an explanation. Experiments 4 and 5 examine the influence of statistical relevance in this regard, both for numerical and for visual representation of the statistical information.

Clarifying the respective impact of prior credibility and statistical relevance on judgments of explanatory power matters to another central topic in the philosophy and psychology of explanation: *abductive reasoning* (Salmon, 1989; Lipton, 2004; Douven, 2011; Schupbach, 2017). When people engage in abductive reasoning, they rely on explanatory considerations to justify the conclusion that a certain hypothesis is true. Specifically, people often infer the truth of that hypothesis H1 from a pool of candidate hypotheses H1, H2, …, Hn, that best explains available evidence E (Thagard, 1989; Douven, 2011). However, whether “best explains” consists in high statistical relevance, generalizability, provision of a plausible cause or some other explanatory virtue remains controversial (Van Fraassen, 1989; Okasha, 2000; Lipton, 2001, 2004; Douven and Schupbach, 2015). Moreover, given the numerous biases in probabilistic reasoning (Kahneman and Tversky, 1982; Hahn and Harris, 2014), it is not clear whether and how statistical relevance will affect explanatory judgment.

In summary, bringing together different strands of research from philosophy and psychology, our study asks: How do the credibility, causal framing, statistical relevance, and perceived generalizability of a hypothesis influence judgments of explanatory power?

The pattern of our experimental findings supports the hypothesis that the prior credibility of a causal explanation plays a central role in explanatory reasoning: first, because of the presence of strong main effects on judgments of explanatory power, and second, because of the gate-keeping role it had for other factors. Highly credible explanations were not susceptible to causal framing effects, which may lead astray explanatory judgment. Instead, highly credible hypotheses were sensitive to the effects of factors which are usually considered relevant from a normative point of view: perceived generalizability of an explanation, and its statistical relevance, operationalized as the strength of association between two relevant properties.

These results advance current literature in the philosophy and psychology of explanation in three ways. First, our results yield a more nuanced understanding of the determinants of judgments of explanatory power, and the interaction between these factors. Second, they show the close relationship between prior beliefs and explanatory power. Third, they elucidate the nature of abductive reasoning.

## Overview of the experiments and pre-tests

We conducted five experiments, where we systematically examined the influence of the possible determinants of explanatory judgment: prior credibility, causal framing, perceived generalizability, and statistical relevance. To warrant the validity of the experimental material, we conducted a series of pre-studies, where participants evaluated different levels of causal framing, credibility, and generalizability. Materials which corresponded to high, low, and neutral levels of these three factors were implemented in the vignettes of our five experiments, either as independent variables or as control variables. Material evaluation and main experiments were both conducted online on Amazon Mechanical Turk, utilizing the Qualtrics Survey Software. We only allowed MTurk workers with an approval rate >95% and with a number of HITs approved >5,000 to submit responses. Instructions and material were presented in English. None of the participants took part in more than one experiment.

### Causal framing

In a pre-study, a sample of *N* = 44 participants (mean age 30.5 years, *SD* = 7.3, 28 male) from America (*n* = 27) and other countries rated eight brief statements, expressing relations between two variables X and Y of the type “X co-occurs with Y”; “X is associated with Y,” and so on (see Appendix A in Supplementary Material for the complete list of statements). The statements were presented in an individually randomized order to the participants; only one statement was visible at a time; and going back to previous statements was not possible. The participants judged how strongly they agreed or disagreed that a certain statement expressed a causal relation between X and Y. Judgments were collected on a 7-point scale with the options: “I strongly disagree” (−3), “I disagree,” “I slightly disagree,” “I neither agree nor disagree” (0), “I slightly agree,” “I agree,” “I strongly agree” (3)^1^. Based on participants' ratings, we selected three types of statements for our main experiments: statements with a neutral causal framing (“X co-occurs with Y”), with a weak causal framing (“X is associated with Y”), and with a strong causal framing (“X leads to Y” and “X causes Y”) (Table 1).

**Table 1:** Wordings that were perceived to express weak, neutral, and strong causal framing of the relationship between an explanans (X) and an explanandum (Y).

**Causal Framing**	**Framing of the hypothesis**
Weak	X is associated with Y
Neutral	X co-occurs with Y
Strong	X causes Y^2^
Strong	X leads to Y

*According to the ratings observed in the pre-study, “X causes Y” and “Y leads to Y” express causal relations to an equal extent*.

### Prior credibility

We identified the prior credibility of different hypotheses by asking a new sample of *N* = 42 participants (mean age 30.7 years, *SD* = 7.5, 16 male) from America (*n* = 29) and other countries to rate a list of 24 statements (Appendix A in Supplementary Material). Participants judged how strongly they disagreed or agreed that a certain hypothesis was credible. For all hypotheses, we used the phrasing “…co-occurs with…” to avoid the influence of causal framing^2^. Based on participants' ratings (see Appendix A in Supplementary Material), we selected four statements to use in our main experiments: two were highly credible, the other two were highly incredible (Table 2).

**Table 2:** The four hypotheses rated as least credible and as most credible.

**Credibility**	**Hypothesis**
Low	Eating pizza co-occurs with immunity to flu.
Low	Drinking apple juice co-occurs with anorexia.
High	Well-being co-occurs with frequent smiling.
High	Consuming anabolic steroids co-occurs with physical strength.

### Generalizability

We conducted a pre-study in order to determine how the description of the sample used in a scientific study influenced the perceived generalizability of the study's results; that is, people's perception that a given study's result applies to many individuals in the general population beyond the sample involved in the study. This pre-study included two questionnaires, which were administered to two different groups of participants. One questionnaire presented descriptions of the samples used in scientific studies, which varied with regard to the *number* of people involved. The other questionnaire presented sample descriptions that varied with regard to the *type* of people in the sample. The statements were presented in an individually randomized order to the participants. Only one statement was visible at a time, and going back to previous statements was not possible.

Forty-two participants (mean age 33.5 years, *SD* = 10.8, 27 male) from America (*n* = 38) and other countries were presented with a list of six brief statements about a sample of a particular number of participants, e.g., “The study investigates 5 people”; “The study investigates 500 people” (see Appendix A in Supplementary Material for the complete list of items). We found that the perceived generalizability of a study increased with the number of people in the sample of the study.

A new group of *N* = 41 participants (mean age 33.0 years, *SD* = 9.7, 26 male) from America (*n* = 36) and other countries was presented with a list of nine brief statements about samples of particular types of people, e.g., “The study investigates a group of people who sit in a park”; “The study investigates a group of people who work at a university” (see Appendix A in Supplementary Material for the complete list of items). However, focusing on the *number* instead of the *type* of people in the sample allowed for a neater distinction between narrowly and widely generalizable results. Therefore, we characterized perceived generalizability as a function of the number of participants in the main vignettes of the experiment (see Table 3)^3^.

**Table 3:** Ratings of the generalizability of studies in the pre-tests, dependent of the number of people in the sample.

**Generalizability**	**Description**
Narrow	The study investigates five people.
Medium	The study investigates 240 people.
Wide	The study investigates 10,000 people.

### Vignettes of the main experiment

All experiments were performed, using a 2 × 2 (within-subject) design with explanatory power as dependent variable and prior credibility of the hypothesis being one of the independent variables. The other independent variable was either causal framing, generalizability, or statistical relevance of the reported research study.

Participants were presented with four short reports about fictitious research studies. Two of these reports involved highly credible hypotheses, the other two reports involved incredible hypotheses. Two reports showed a high level of the other independent variable (causal framing/generalizability/statistical relevance), while the other two reports showed a low level of that variable. To account for the possible influence of the content of a particular report, the allocation of low and high levels of that variable was counterbalanced to the credibility conditions across the items, leading to two versions of each experiment.

Each vignette in our experiments followed the same format, including a headline and five sentences. The headline stated the hypothesis, the first sentence introduced the study, the second sentence described the sample size, the third sentence reported the results of the study, and the fourth sentence reported factors controlled by the researchers. The final sentence presented a brief conclusion, essentially restating the hypothesis. We now present a sample vignette for a study that investigates the link between anabolic steroids and physical strength. For details of the vignettes in the individual experiments, see Appendices B–D in Supplementary Material.

#### Consuming anabolic steroids leads to physical strength

A recent study by university researchers investigated the link between consuming anabolic steroids and physical strength. The researchers studied 240 persons. The level of physical strength was higher among participants who regularly consumed anabolic steroids than among the participants who did not regularly consume anabolic steroids. Family health history, age, and sex, which were controlled by the researchers, could not explain these results. The study therefore supports the hypothesis that consuming anabolic steroids leads to physical strength.

**Figure 1:**
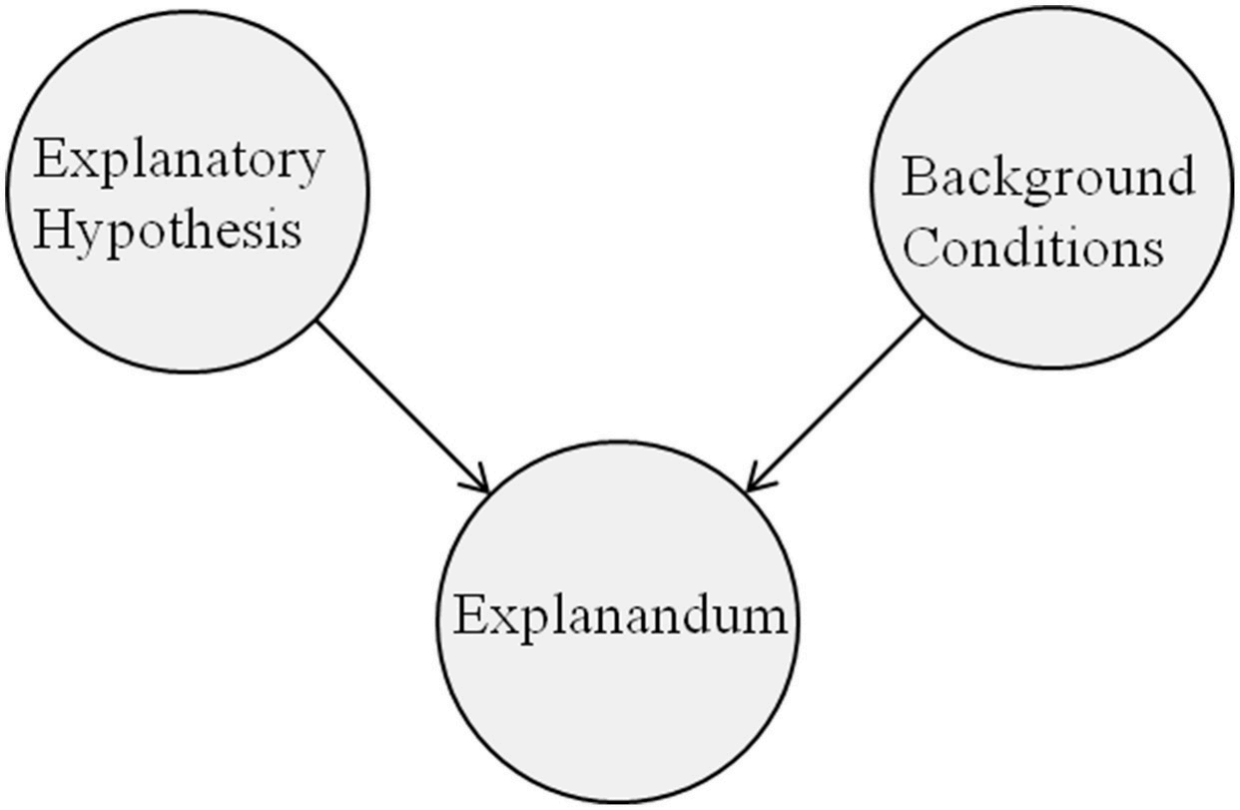
A schematic representation of the components of an explanation. In our vignettes, the explanatory hypothesis postulates a causal relationship (e.g., “consuming anabolic steroids leads to physical strength”); the *explanandum* states the result of the study (e.g., higher rate of physical strength in the treatment group).

In all experiments, we varied the level of prior credibility of a hypothesis. In Experiments 1 and 2, we also varied the causal framing and interchanged “leads to” with “causes” and “is associated with,” while we kept generalizability at its control value (*N* = 240) and did not provide information about statistical relevance. In Experiment 3, we varied the sample size (= generalizability) and controlled for causal framing by using the predicate “co-occurs with” in the headline and the conclusion. Finally, in Experiments 4 and 5, we varied the levels of statistical relevance (= the frequency of a causal effect in the treatment and in the control group) while controlling for causal framing (“X co-occurs with Y”) and generalizability (*N* = 240). See Figure 1 for a schematic representation of the components of an explanation. In this picture, two of our four independent variables are properties of the explanatory hypothesis (prior credibility, causal framing) while generalizability of the results pertains to the *explanandum* (= the study results) in relation to the background conditions (= study design and population). Similarly, statistical relevance expresses a property of the *explanandum* with respect to the explanatory hypothesis.

Participants were asked to rate our dependent variable: the explanatory power of the stated hypothesis for the results of the study. Specifically, participants were asked to indicate on a Likert scale the extent to which they agreed or disagreed that a target hypothesis explained the experimental results presented in a vignette. A Likert scale was employed for its simplicity in use and understanding, although responses are not obviously translated into numerical values that may pick out different degrees of explanatory power. Given that we were interested in the power of explanations *relative* to variation in the values of possible determinants of explanatory power, we expected that an “agreement scale” to be sufficient to test the relative impact of different factors.^4^

## Experiments 1 and 2. credibility × causal framing

Two-hundred-three participants (mean age 34.7 years, *SD* = 10.5; 121 male) from America (*n* = 130), India (*n* = 67) and other countries completed Experiment 1 for a small monetary payment. A new sample of two-hundred-eight participants (mean age 34.56 years, *SD* = 9.97; 124 male) from America (*n* = 154), India (*n* = 43), and other countries completed Experiment 2 for a small monetary payment.

### Design and material

In both experiments, participants were presented with four short reports about fictitious research studies along the lines of the above vignette. Across vignettes, we manipulated the causal framing of the relationship between hypothesis and evidence as well as the choice of the hypothesis (credible vs. incredible). Generalizability was controlled for by setting it to its medium value (240 participants). Two of the four reports involved highly credible hypotheses, the other two reports involved incredible hypotheses. Similarly, two of these reports used weak causal framing (Experiments 1 and 2: “X is associated with Y”) while the other two reports used strong causal framing (Experiment 1: “X leads to Y,” Experiment 2: “X causes Y”). In other words, Experiment 1 used implicit causal language and Experiment 2 used explicit causal language, while the experiments were, for the rest, identical with respect to design, materials, and procedure.

To account for the possible influence of the content of a particular report, we counterbalanced the allocation of weak and strong causal framing conditions to the credibility conditions across the items, and created two versions of the experiments: Version A and B (see Appendix B in Supplementary Material for details). The order of reports was individually randomized for each participant.

### Procedure

Participants judged each report in terms of the explanatory power of the hypothesis it described. Specifically, participants considered the statement: “The researchers' hypothesis explains the results of the study,” and expressed their judgments on a 7-point scale with the extremes (−3) “I strongly disagree” and (3) “I strongly agree,” and the center pole (0) “I neither disagree nor agree.”

### Analysis and results

**Figure 2:**
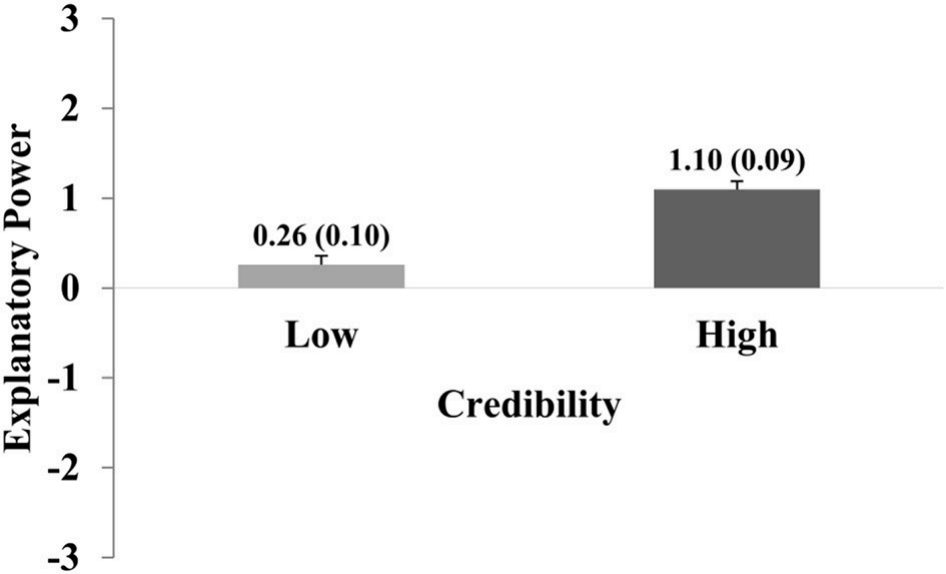
The graph shows explanatory power ratings for credible and incredible statements in Experiment 1. Ratings were significantly higher for credible as opposed to incredible statements. Error bars show standard errors of the mean and are also expressed numerically, in parentheses next to the mean value.

Separate two-way ANOVAs were calculated for Experiments 1 and 2, with the factors Credibility (low, high) and Causal Framing (weak, strong). ANOVA of Experiment 1 (implicit causal language) revealed a main effect of Credibility, *F*_(1, 202)_ = 84.5; *p* < 0.001; η^2^_*part*_ = 0.30. There was no main effect of Causal Framing (*p* = 0.37), and no interaction (*p* = 0.08). Pair-wise comparisons showed that incredible hypotheses were rated significantly lower than credible hypotheses, independently of the value of Causal Framing [incredible hypotheses: *M* = 0.26; *SEM* = 0.10; credible hypotheses: *M* = 1.14; *SEM* = 0.09; *t*-test: *t*_(202)_ = −9.2; *p* < 0.001; *d* = 0.67]. See Figure 2. The results of Experiment 1 therefore indicate that the prior credibility of a hypothesis was a strong predictor of judgments of explanatory power. Instead, framing a hypothesis with implicit causal language did not have effects on explanatory judgment.

**Figure 3:**
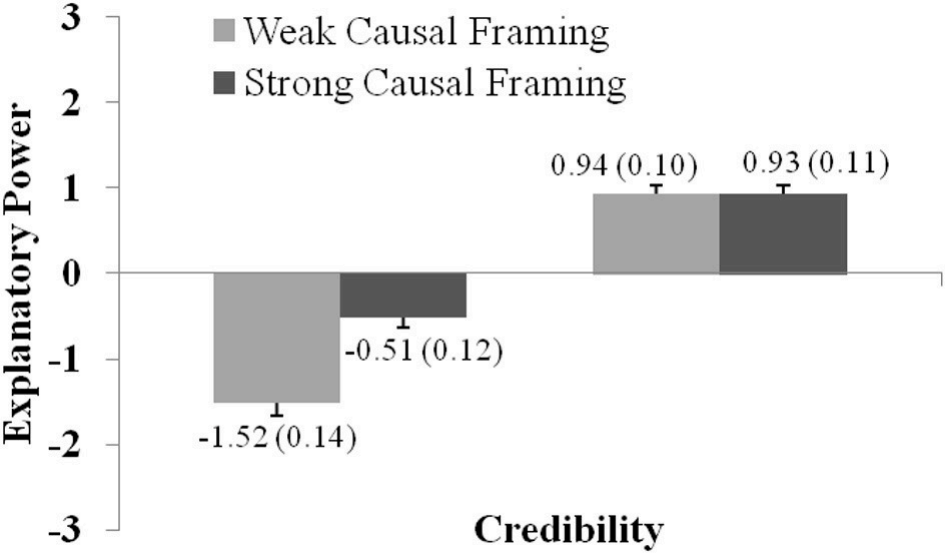
The graph shows how explanatory power ratings vary with regard to Credibility and Causal Framing (as presented in Experiment 2). Ratings were significantly higher for statements with high compared to low Credibility, and for statements with strong compared to weak Causal Framing. The graph shows the (significant) interaction between both factors. Error bars show standard errors of the mean and are also expressed numerically, in parentheses next to the mean value.

ANOVA of Experiment 2 (explicit causal language) revealed main effects of Credibility [*F*_(1, 207)_ = 286.9; *p* < 0.001; η^2^_part_ = 0.58] and Causal Framing, *F*_(1, 207)_ = 31.0; *p* < 0.001; η^2^_*part*_ = 0.13, as well as a significant interaction Credibility × Causal Framing, *F*_(1, 207)_ = 37.6; *p* < 0.001; η^2^_*part*_ = 0.15. Figure 3 shows the effect sizes and the interaction between both factors as well as the relevant descriptives.

The results of Experiment 2 therefore confirm that the prior credibility of a hypothesis is a strong predictor of judgments of the hypothesis' explanatory power. Incredible hypotheses received relatively lower explanatory power ratings, while credible hypotheses received relatively higher ratings, *t*_(207)_ = −16.936; *p* < 0.001; *d* = 1.347. The results also showed that explicit causal framing can increase ratings of explanatory power, but only for incredible hypotheses, *t*_(207)_ = −7.253; *p* < 0.001; *d* = 0.545. While this effect may lead explanatory judgment astray, in most practical cases of explanatory reasoning, people are interested in the explanatory power of hypotheses which they find, at least to a certain extent, credible. As Figure 3 shows, there was no effect of causal framing on explanatory power in this important case.

All in all, the observed patterns in both experiments confirm that the prior credibility of a hypothesis plays a gate-keeping-role in explanatory reasoning: only credible causal hypotheses qualify as explanatorily valuable. Implicit or explicit causal framing plays a small to negligible role in influencing judgments of explanatory power.

## Experiment 3: credibility × generalizability

### Participants

Two-hundred-seven participants (mean age 33.4 years, *SD* = 9.1; 123 male) from America (*n* = 156), India (*n* = 37) and other countries completed Experiment 3 for a small monetary payment.

### Design and material

The experiment resembled Experiments 1 and 2. Four vignettes, each of which included a headline and five sentences, presented credible and incredible hypotheses. The relation between hypothesis and evidence was expressed by using the causally neutral wording “X co-occurs with Y.” The critical manipulation concerned the sample descriptions used in the vignettes, which expressed either narrowly or widely generalizable results. For narrowly generalizable results, the second sentence of a report indicated that the sample of the study encompassed around 5 people (e.g., “The researchers studied 6 people”). For widely generalizable results, the sample included about 10,000 people (*wide* generalizability condition, e.g., “The researchers studied 9,891 people”).

To control for the possible influence of the content of a particular report, we counterbalanced the allocation of narrow and wide generalizability conditions to the credibility conditions across the items, and created two versions of the experiment (see Appendix C in Supplementary Material for detailed information). The order in which reports were presented to the participants was individually randomized for each participant.

### Procedure

Participants were asked to carefully assess each report with regard to Explanatory Power. Participants' ratings were collected on 7-point scales, with the extreme poles (−3) “I strongly disagree” and (3) “I strongly agree,” and the center pole (0) “I neither disagree nor agree.”

### Analysis and results

The ratings were analyzed with a two-way ANOVA with the factors Credibility (low, high) and Generalizability (narrow, wide). ANOVA revealed significant main effects of Credibility, *F*_(1, 206)_ = 83.830; *p* < 0.001; η^2^_part_ = 0.289; and Generalizability, *F*_(1, 206)_ = 29.593; *p* < 0.001; η^2^_part_ = 0.126, and no interaction Credibility × Generalizability (*p* = 0.085, n.s.).

**Figure 4:**
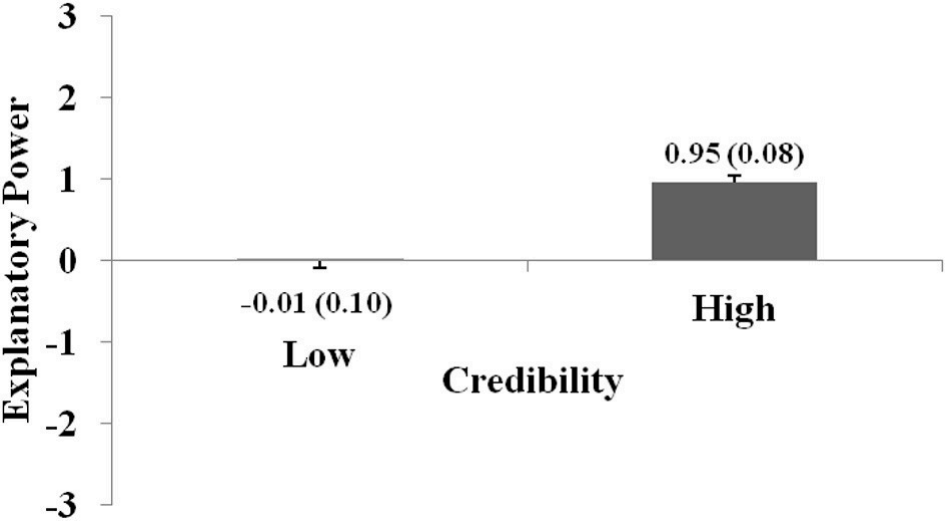
The graph shows how explanatory power ratings vary with regard to Credibility. Ratings were significantly higher for statements with high compared to low Credibility. The graph shows the main effect for this factor. Error bars show standard errors of the mean and are also expressed numerically, in parentheses next to the mean value.

**Figure 5:**
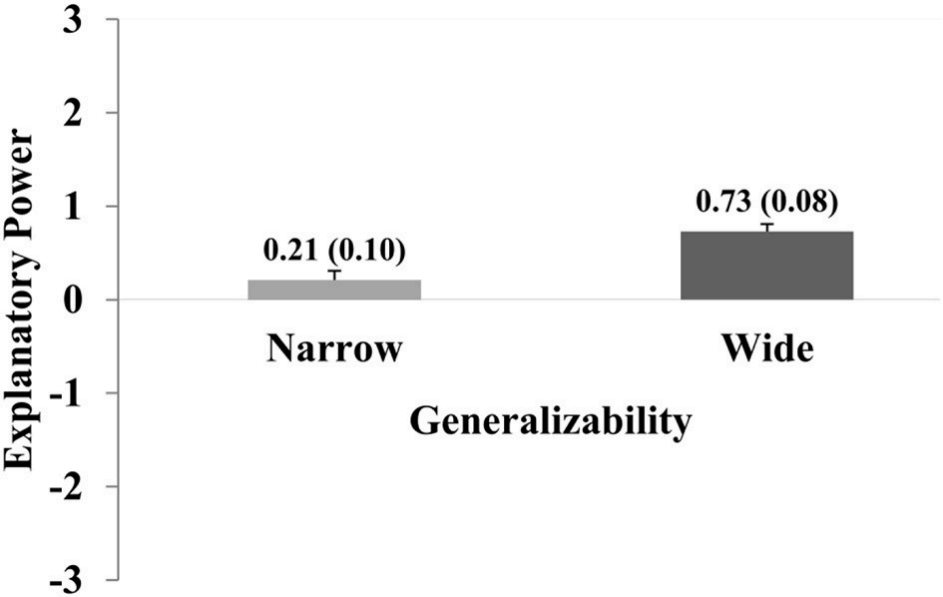
The graph shows how explanatory power ratings vary with regard to Generalizability. Ratings were significantly higher for statements with high compared to low Generalizability. The graph shows the main effect for this factor. Error bars show standard errors of the mean and are also expressed numerically, in parentheses next to the mean value.

As with Experiments 1 and 2, credible hypotheses achieved significantly higher ratings than incredible hypotheses [incredible hypotheses: *M* = −0.01; *SEM* = 0.10; credible hypotheses: *M* = 0.95; *SEM* = 0.08; *t*-test: *t*_(206)_ = −9.2; *p* < 0.001; *d* = 0.72]. Furthermore, reports with wide generalizability achieved significantly higher ratings compared to reports with narrow generalizability [narrow: *M* = 0.21; *SEM* = 0.10; credible hypotheses: *M* = 0.73; *SEM* = 0.08; *t*_(206)_ = −5.4; *p* < 0.001; *d* = 0.40]. Figures 4 and 5 show the main effects for both variables.

## Experiments 4 and 5: credibility × statistical relevance

Experiments 4 and 5 examined in what way probabilistic information influences explanatory judgments and how statistical information is taken into account for credible vs. incredible hypotheses. Experiment 4 presented the statistical information numerically, Experiment 5 presented it visually.

### Participants

Two-hundred-three participants (mean age 34.7 years, *SD* = 9.5; 122 male) from America (*n* = 168), India (*n* = 15), and other countries completed Experiment 4 for a small monetary payment. A new sample of *N* = 208 participants (mean age: 36.0 years, *SD* = 19.7; 133 male), from America (*n* = 122), India (*n* = 69), and other countries completed Experiment 5 for a small monetary payment.

### Design and material

**Figure 6:**
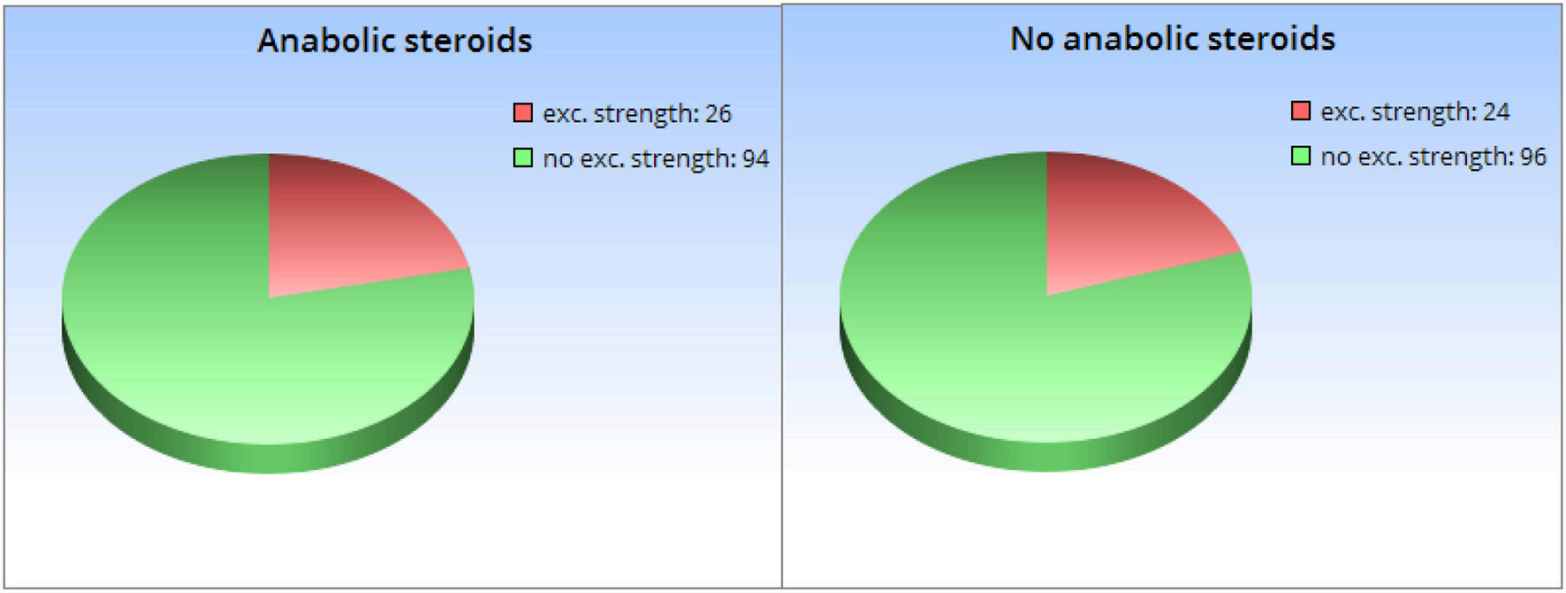
Visual representation of statistical information of the fictitious research groups as provided in Experiment 5.

The experiments resembled the previous ones. The four vignettes presented credible and incredible hypotheses. The sample descriptions in the vignettes were chosen such that both, generalizability and causality, were perceived as “neutral,” according to the results of our pre-study. This meant that we opted for a medium-sized population sample of 240 persons (like in Experiments 1 and 2) and the wording “X co-occurs with Y” (like in Experiment 3). The novel manipulation was implemented in the part of the vignette where the results of the study are reported. This part now included statistical information. In a case of weak statistical relevance, the frequency of the property of interest was almost equal in the treatment and control group, e.g.,: “Among the participants who regularly consumed anabolic steroids, *26 out of 120 (* = *22%)* exhibited an exceptional level of physical strength. Among the participants who did not regularly consume anabolic steroids, *24 out of 120 (* = *20%)* exhibited an exceptional level of physical strength.” For strong statistical relevance, there was a notable difference in the frequency of the property of interest, e.g.,: “Among the participants who regularly consumed anabolic steroids, *50 out of 120 (* = *42%)* exhibited an exceptional level of physical strength. Among the participants who did not regularly consume anabolic steroids, *7 out of 120 (* = *6%)* exhibited an exceptional level of physical strength.” While Experiment 4 represented the statistical information numerically like in the previous sentences, Experiment 5 stated the same absolute numbers and replaced the accompanying percentages with two pie charts (see Figure 6).

As in the previous experiments, we counterbalanced the allocation of the weak statistical relevance and strong statistical relevance conditions across the items, and created two versions of each experiment (see Appendix D in Supplementary Material for detailed information). The order of reports was individually randomized for each participant.

### Procedure

Participants were asked to carefully assess each report with regard to Explanatory Power. Again, the ratings of the participants were collected on 7-point scales, with the extreme poles (−3) “I strongly disagree” and (3) “I strongly agree,” and the center pole (0) “I neither disagree nor agree.”

### Analysis and results

Separate two-way ANOVAs were calculated for Experiments 4 and 5, with the factors Credibility (low, high) and Statistical Relevance (weak, strong). ANOVA of Experiment 4 revealed significant main effects of Credibility, *F*_(1, 202)_ = 65.3; *p* < 0.001; η^2^_*part*_ = 0.24 and Statistical Relevance, *F*_(1, 202)_ = 74.2; *p* < 0.001; η^2^_*part*_ = 0.27, and a significant interaction Credibility × Statistical Relevance, *F*_(1, 202)_ = 47.7; *p* < 0.001; η^2^_*part*_ = 0.19.

**Figure 7:**
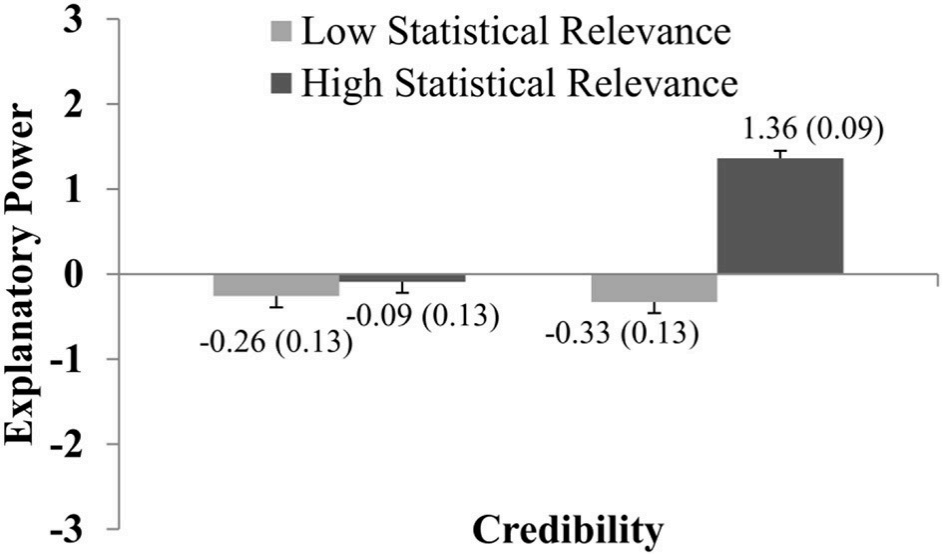
The graph shows how explanatory power ratings vary with regard to Credibility and Statistical Relevance (as presented in Experiment 4). Ratings were significantly higher for statements with high compared to low Credibility, and for statements with high compared to low Statistical Relevance. The graph shows the (significant) interaction between both factors. Error bars show standard errors of the mean and are also expressed numerically, in parentheses next to the mean value.

Figure 7 shows the effect sizes and the interaction between both factors as well as the relevant descriptive statistics. Relatively high levels of explanatory power were only achieved for highly credible hypotheses and high statistical relevance. The other conditions roughly led to the same explanatory power ratings (*p'*s > 0.25). This suggests that both factors act as a gate-keeper in explanatory reasoning: if they take their low values, no hypothesis can be rated as explanatorily powerful. On the other hand, if both conditions are satisfied, the effect is very pronounced, *t*_(202)_ = −11.82; *p* < 0.001; *d* = 0.89 (comparison of high credible reports with weak and strong statistical relevance).

Similar results were obtained for Experiment 5. ANOVA of Experiment 5 revealed significant main effects of Credibility, *F*_(1, 207)_ = 38.2; *p* < 0.001; η^2^_*part*_ = 0.16, and Statistical Relevance, *F*_(1, 207)_ = 152.5; *p* < 0.001; η^2^_*part*_ = 0.42, and a significant interaction Credibility × Statistical Relevance, *F*_(1, 207)_ = 47.4; *p* < 0.001; η^2^_*part*_ = 0.10.

**Figure 8:**
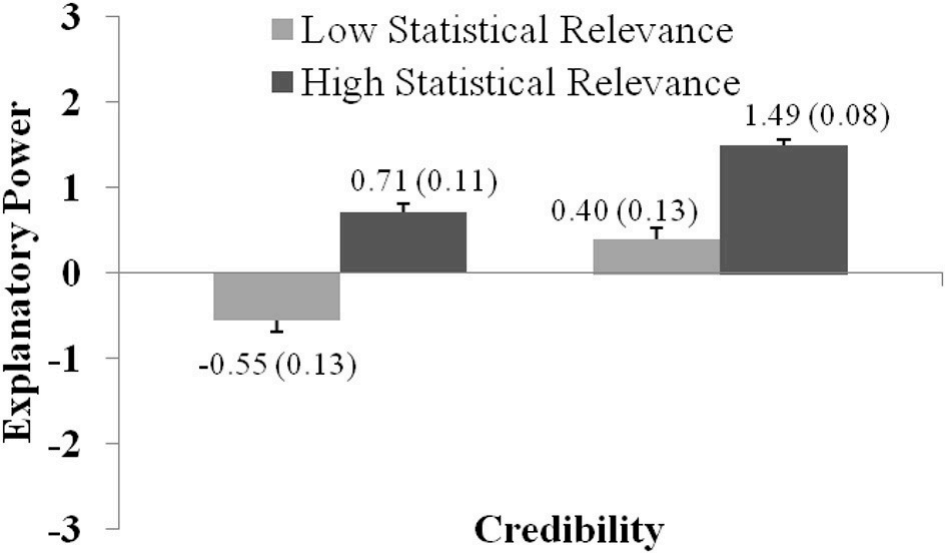
The graph shows how explanatory power ratings vary with regard to Credibility and Statistical Relevance (as presented in Experiment 5). Ratings were significantly higher for statements with high compared to low Credibility, and for statements with high compared to low Statistical Relevance. The graph shows the (significant) interaction between both factors. Error bars show standard errors of the mean and are also expressed numerically, in parentheses next to the mean value.

Figure 8 shows the effect sizes and the interaction between both factors as well as the relevant descriptives. We found a slightly different interaction pattern than in Experiment 4. Again, both variables have to take their high values for a hypothesis to be rated as explanatorily powerful. However, we also see that the gate-keeping role of both variables is weaker than in the case where statistical information was only presented numerically: *t*_(207)_ = −8.85; *p* < 0.001; *d* = 0.71 (comparison of low credible reports with weak and strong statistical relevance) and *t*_(207)_ = −13.19; *p* < 0.001; *d* = 0.69 (comparison of high credible reports with weak and strong statistical relevance). Either variable taking its high value suffices for a judgment of relatively high explanatory power. Like in Experiment 4, the level of explanatory power was by far the highest in the condition where both credibility and statistical relevance were high. These findings also resonate well with the more normatively oriented literature on statistical explanation which sees explanatory power as an increasing function of the surprisingness of the explanandum (Hempel, 1965; Salmon, 1971; Schupbach and Sprenger's, 2011; Crupi and Tentori, 2012). Weak statistical associations are less surprising than strong associations, therefore an adequate explanatory hypothesis (i.e., a hypothesis that postulates the right sort of causal relationship) is more powerful in the latter case, ceteris paribus.

## Discussion

We examined the impact of four factors—prior credibility, causal framing, perceived generalizability, and statistical relevance—on judgments of explanatory power. In a series of five experiments, we varied both the subjective credibility of an explanation and one of the other factors: causal framing, generalizability, and statistical relevance (both with numeric and with visual presentation of the statistics). In Experiments 1 and 2 we found that the impact of causal language on judgments of explanatory power was small to negligible. Experiment 3 showed that explanations with wider scope positively affected judgments of explanatory power. In Experiments 4 and 5, we found that explanatory power increased with the statistical relevance of the explanatory hypothesis for the observed evidence.

Across all experiments, we found that the prior subjective credibility of a hypothesis had a striking effect on how participants assessed explanatory power. In particular, the credibility of an explanatory hypothesis had an important gate-keeping function: the impact of statistical relevance on explanatory power was more significant when credibility was high. On the other hand, the high credibility of a hypothesis controlled for the potentially misleading effect of causal framing on explanatory judgment.

This pattern of findings is consistent with existing psychological research demonstrating that people resist endorsing explanatory hypotheses that appear unnatural and unintuitive, given their background common-sense understanding of the physical and of the social world (Bloom and Weisberg, 2007). Our findings are also consistent with the idea that stable background personal ideologies (often referred to as “worldview”) can reliably predict whether people are likely to reject well-confirmed scientific hypotheses (Lewandowsky et al., 2013; Colombo et al., 2016b). So, scientific hypotheses that are inconsistent with our prior background beliefs are likely to be judged as implausible, and may not be endorsed as good explanations unless they are supported by extra-ordinary evidence gathered by some trustworthy source. On the other hand, for hypotheses that fit our prior, background belief or ideology, we often focus on information that, if the candidate explanatory hypothesis is true, would boost its goodness (Klayman and Ha, 1987).

This kind of psychological process of biased evidence evaluation and retention bears a similarity to the properties of incremental measures of confirmation called *Matthew properties* (Festa, 2012). According to confirmation measures presenting Matthews properties, an equal degree of statistical relevance leads to higher (incremental) confirmation when the hypothesis is already credible than when it is incredible. The same was observed in our experiment, where the effect of statistical relevance on different dimensions of explanatory power was much more pronounced for credible than for incredible hypotheses. Moreover, the highest ratings of explanatory power, across different experiments, were achieved when, in addition to a credible hypothesis, the report was perceived as widely generalizable, its statistical relevance for the observed results was high. Only in those cases, a relatively higher degree of explanatory power was achieved. This confirms that those factors play a crucial role in explanatory reasoning: the more an explanation is perceived to be credible, statistically relevant and widely generalizable, the higher its perceived explanatory power.

The interplay we observed between statistical relevance, prior credibility, and explanatory power is also relevant to understanding the nature of abductive reasoning. In abductive reasoning, explanatory considerations are taken to boost the credibility of a target hypothesis while inducing a sense of understanding (Lipton, 2004). We showed that high prior credibility may insulate an explanation from causal framing effects. However, when an explanation is surprising or otherwise incredible, like most of the explanations that feature in newspapers and magazines, causal framing may increase the perceived power of the explanation, producing a deceptive sense of understanding (Rozenblit and Keil, 2002; Trout, 2002). Moreover, while previous studies investigated the role of simplicity and coherence in abductive reasoning (Lombrozo, 2007; Koslowski et al., 2008; Bonawitz and Lombrozo, 2012), our results extend this body of literature by showing how the generalizability of a hypothesis and its statistical relevance influence the perceived quality of an explanation.

Overall, our experiments show that explanatory power is a complex concept, affected by considerations of prior credibility of a (causal) hypothesis, generalizability and statistical relevance. These factors also figure prominently in (normative) philosophical theories of explanation. For instance, the D-N model (Hempel, 1965) stresses the generality of the proposed explanation, the causal-mechanical account (Woodward, 2003) requires a credible causal mechanism, and statistical explanations are usually ranked according to their relevance for the observed evidence (Salmon, 1970; Schupbach and Sprenger's, 2011).

On the other hand, the multitude of relevant factors in explanatory judgment explains why it has been difficult to come up with a theory of abductive inference that is both normatively compelling and descriptively accurate: after all, it is difficult to fit quite diverse determinants of explanatory judgment into a single unifying framework. In that spirit, we hope that our results will promote an interdisciplinary conversation between empirical evidence and philosophical theorizing, and about the “prospects for a naturalized philosophy of explanation” in particular (Lombrozo, 2011; Colombo, 2017; Schupbach, 2017).

## Ethics statement

This study was carried out in accordance with the recommendations of the APA Ethical Principles of Psychologists and in accordance with the Declaration of Helsinki. Prior to the experiment, participants were debriefed about the purpose and the aim of the study, and presented with the informed consent document, including instructions that by starting the questionnaire, they indicate consent to participate in the study.

## Author contributions

The paper is fully collaborative. While MC and JS were mainly responsible for the theoretical framing and LB for the statistical analysis, each author worked on each section of the paper.

### Conflict of interest statement

The authors declare that the research was conducted in the absence of any commercial or financial relationships that could be construed as a potential conflict of interest.

## Footnotes

^1^Only the verbal labels (from “I strongly agree” through “I strongly disagree” were visible for the participants. The assigned values (-3 through 3) were unknown to participants.

^2^Different content as well as differences in causal framing may obviously change the credibility of a given hypothesis. One reason why causal language changes the credibility of particular hypotheses is that *co-occurrence* and *association* are symmetric relations, whereas *causation* is an asymmetric relation. For example, “Liver cancer co-occurs with drinking alcohol” and “Drinking alcohol co-occurs with liver cancer” are both equally credible. Instead, “Liver cancer causes drinking alcohol.” is much less credible than “Drinking alcohol causes liver cancer.”

^3^From a statistical point of view, a larger sample size need not actually affect the generalizability of a result. Sample size affects, instead, *confidence levels* and *margins of error, power* and *effect sizes*.

^4^As a reviewer helpfully pointed out, we might have asked participants the direct question: “How well in your opinion does the hypothesis H explain results E?” In principle, people can in fact strongly agree that a given hypothesis H explains the results of a study E, while they can hold at the same time that H is not a very good explanation of E. This type of question may have given us a clearer picture of participants' absolute judgments of the explanatory power of H. Yet, this was unnecessary for the purpose of our study, that is, for determining whether variation in the value of various factors bring about significant changes in judgments of explanatory power.
