# Supplementary material for: Determinants of Judgments of Explanatory Power: Credibility, Generality, and Statistical Relevance
Source: Front Psychol. 2017 Sep 4;8:1430. doi: 10.3389/fpsyg.2017.01430 (PMC5591454; doi:10.3389/fpsyg.2017.01430)
Supplement: Supplementary file 1 [file Appendix.docx]

**Appendix A (Pre-tests)**

A.1 List of statements expressing relationships between an explanans (X) and an explanandum Y) presented in the pre-study on Causal Framing.

| \| **Statements** \| **Ratings**  **Mean (Standard Deviation)** \| \| --- \| --- \| \| X prevents Y. \| -0.59 (1.98) \| \| X co-occurs with y. \| 0.09 (1.60) \| \| X predicts Y. \| 0.25 (1.57) \| \| X is associated with Y. \| 0.70 (1.83) \| \| X promotes Y. \| 0.80 (1.46) \| \| X is correlated with Y. \| 0.86 (1.64) \| \| X causes Y. \| 1.02 (2.01) \| \| X leads to Y. \| 1.23 (1.67) \| |  |
| --- | --- | --- | --- | --- | --- | --- | --- | --- | --- | --- | --- | --- | --- | --- | --- | --- | --- | --- | --- |

A.2 List of hypotheses, presented to the participants (N = 42) of the pre-study on Credibility, and mean ratings (and standard deviation), collected on 7-point scales with the options: "I strongly disagree" (-3), "I disagree", "I slightly disagree", "I neither agree nor disagree" (0), "I slightly agree", "I agree", "I strongly agree" (3).

| **Hypotheses** | **Ratings:**  **Mean (Standard Deviation)** |
| --- | --- |
| Eating pizza co-occurs with immunity to flu. | -1.95 (1.45) |
| Drinking apple juice co-occurs with anorexia. | -1.86 (1.28) |
| Breast feeding co-occurs with hair loss in the baby. | -1.83 (1.46) |
| Vegetarianism co-occurs with aggressiveness. | -1.76 (1.36) |
| Helpfulness co-occurs with blond hair. | -1.79 (0.23) |
| Exercising co-occurs with frequent headache. | -1.45 (1.61) |
| Kleptomania co-occurs with sexual deprivation. | -1.33 (1.53) |
| Eating crab co-occurs with good eyesight. | -1.12 (1.58) |
| Attending religious services co-occurs with positive mood. | 0.14 (1.69) |
| Drinking coffee co-occurs with higher blood pressure. | 0.43 (1.73) |
| Vandalism co-occurs with low self-esteem. | 0.52 (1.15) |
| Low interest rates co-occur with a high number of newly built houses. | 0.69 (1.65) |
| Professional success co-occurs with parental income above $ 100,000/year. | 0.74 (1.50) |
| Having breakfast co-occurs with a healthy body mass index. | 0.79 (1.22) |
| Rainy days co-occur with birds breeding. | 0.79 (1.62) |
| Eating hot dogs co-occurs with obesity. | 0.83 (1.50) |
| Drinking whisky co-occurs with liver cancer. | 0.90 (1.65) |
| Smoking cannabis co-occurs with drowsiness. | 1.10 (1.27) |
| Well-being co-occurs with frequent smiling. | 1.14 (1.46) |
| Consuming anabolic steroids co-occurs with physical strength. | 1.21 (1.86) |

**A.3 List of sample description statements presented in the pre-study on Generalizability**

1. Sample description based on the *number* of participants of a study

| **Statements** | **Ratings: Mean (Standard Deviation)** |
| --- | --- |
| The study investigates 5 people. | -1.88 (1.60) |
| The study investigates 50 people. | -1.05 (1.82) |
| The study investigates 100 people. | -0.43 (1.78) |
| The study investigates 500 people. | 0.55 (1.70) |
| The study investigates 1,000 people. | 0.93 (1.80) |
| The study investigates 10,000 people. | 1.24 (2.05) |

2. Sample description based on the *type* of participants of a study

| **Statements** | **Ratings: Mean (Standard Deviation)** |
| --- | --- |
| The study investigates a group of people who sit in a park. | -0.34 (1.88) |
| The study investigates a group of people who work at a university. | -0.05 (1.97) |
| The study investigates a group of people who attend a religious ceremony. | 0.07 (1.82) |
| The study investigates a group of people who have their number in the telephone book. | 0.12 (2.19) |
| The study investigates a group of people who watch a movie in the cinema. | 0.22 (1.80) |
| The study investigates a group of people who wait for their flight at an airport | 0.27 (1.91) |
| The study investigates a group of people who attend a sports event. | 0.29 (1.82) |
| The study investigates a group of people who shop at a mall. | 0.49 (1.69) |
| The study investigates a group of people who are registered on Facebook. | 0.85 (1.85) |

**Appendix B (Experiment 1 and 2)**

The table shows the allocation of strong (as opposed to weak) causal framing conditions as implemented by the wording "X leads to Y" (Experiment 1) and "X causes Y" (Experiment 2) to the four hypotheses in the two different versions (A and B) of Experiment 1 and 2. In Experiment 1, n = 103 participants completed version A, the remaining participants (N = 100) completed version B. In Experiment 2, N = 103 completed Version A and N = 108 completed version B.

|  | **Experiment 1 and 2** | |
| --- | --- | --- |
| **Credibility** | **Version A** | **Version B** |
| **Low** | Eating pizza *is associated with* immunity to flu.  **Weak Causal Framing** | Eating pizza *leads to/causes^[[1]](#footnote-1)^* immunity to flu.  **Strong Causal Framing** |
| **Low** | Drinking apple juice *leads to/causes* anorexia.  **Strong Causal Framing** | Drinking apple juice *is associated with* anorexia.  **Weak Causal Framing** |
| **High** | Well-being *is associated with* frequent smiling  **Weak Causal Framing** | Well-being *leads to/causes* frequent smiling  **Strong Causal Framing** |
| **High** | Consuming anabolic steroids *leads to/causes* physical strength.  **Strong Causal Framing** | Consuming anabolic steroids *is associated with* physical strength.  **Weak Causal Framing** |

**Appendix C (Experiment 3)**

The table shows the allocation of "narrow" and "wide generalizability" conditions to the four hypotheses in the two different versions (A and B) of Experiment 3. N = 104 participants completed Version A, the remaining participants (N = 104) completed Version B.

|  | **Experiment 3** | |
| --- | --- | --- |
| **Credibility** | **Version A** | **Version B** |
| **Low** | Eating pizza co-occurs with immunity to flu. [...] *The researchers examined 6 persons.*  **Narrow generalizability** | Eating pizza co-occurs with immunity to flu. [...] *The researchers examined 10187 persons.*  **Wide generalizability** |
| **Low** | Drinking apple juice co-occurs with anorexia. [...] *The researchers examined 9891 persons.*  **Wide generalizability** | Drinking apple juice co-occurs with anorexia. [...] *The researchers examined 6 persons.*  **Narrow generalizability** |
| **High** | Well-being co-occurs with frequent smiling. [...] *The researchers examined 10391 persons.*  **Wide generalizability** | Well-being co-occurs with frequent smiling. [...] *The researchers examined 5 persons.*  **Narrow generalizability** |
| **High** | Consuming anabolic steroids co-occurs with physical strength. [...] *The researchers examined 5 persons.*  **Narrow generalizability** | Consuming anabolic steroids co-occurs with physical strength. [...] *The researchers examined 9971 persons.*  **Wide generalizability** |

**Appendix D (Experiment 4 and 5)**

The table below shows the allocation of "low" and "high statistical relevance" conditions to the four hypotheses in the two different versions (A and B) of Experiment 4 and 5. N = 101 participants completed Experiment 4’s version A, the remaining participants (N = 102) completed Experiment 4’s version B. In Experiment 5, N = 106 completed Version A and N = 102 completed version B.

|  | **Experiment 4 and 5** | |
| --- | --- | --- |
| **Credibility** | **Version A** | **Version B** |
| **Low** | Eating pizza co-occurs with immunity to flu. [...] Among the participants who regularly ate pizza, *27 out of 120 (= 23%)* exhibited immunity to flu. Among the participants who did not regularly eat pizza, *25 out of 120 (= 21%)* exhibited immunity to flu.  **Low statistical relevance** | Eating pizza co-occurs with immunity to flu. [...] Among the participants who regularly ate pizza, *48 out of 120 (= 40%)* exhibited immunity to flu. Among the participants who did not regularly eat pizza, *6 out of 120 (= 5%)* exhibited immunity to flu.  **High statistical relevance** |
| **Low** | Drinking apple juice co-occurs with anorexia. [...]Among the participants who regularly drank apple juice, *48 out of 120 (= 40%)* exhibited anorexia. Among the participants who did not regularly drink apple juice, *6 out of 120 (= 5%)* exhibited anorexia.  **High statistical relevance** | Drinking apple juice co-occurs with anorexia. [...]Among the participants who regularly drank apple juice, *26 out of 120 (= 22%)* exhibited anorexia. Among the participants who did not regularly drink apple juice, *24 out of 120 (= 30%)* exhibited anorexia.  **Low statistical relevance** |
| **High** | Consuming anabolic steroids co-occurs with physical strength. [...] Among the participants who regularly consumed anabolic steroids, 26 *out of 120 (= 22%)* exhibited an exceptional level of physical strength. Among the participants who did not regularly consume anabolic steroids, *24 out of 120 (= 20%)* exhibited an exceptional level of physical strength.  **Low statistical relevance** | Consuming anabolic steroids co-occurs with physical strength. [...]  Among the participants who regularly consumed anabolic steroids, *50 out of 120 (= 42%)* exhibited an exceptional level of physical strength. Among the participants who did not regularly consume anabolic steroids, *7 out of 120 (= 6%)* exhibited an exceptional level of physical strength.  **High statistical relevance** |
| **High** | Well-being co-occurs with frequent smiling. [...] Among the participants who reported a high level of well-being, *50 out of 120 (= 42%)* smiled frequently. Among the participants who did not report a high level of well-being, *7 out of 120 (= 6%)* smiled frequently.  **High statistical relevance** | Well-being co-occurs with frequent smiling. [...] Among the participants who reported a high level of well-being, *27 out of 120 (= 23%)* smiled frequently. Among the participants who did not report a high level of well-being, *25 out of 120 (= 21%)* smiled frequently.  **Low statistical relevance** |

1. The phrasing "leads to" was used for high/strong causal items in Experiment 1, and was replaced by the explicit causal wording "causes" in Experiment 2. [↑](#footnote-ref-1)
